# Supplementary material for: Phenotypic Biomarkers of Aqueous Extracellular Vesicles from Retinoblastoma Eyes
Source: Int J Mol Sci. 2024 Oct 30;25(21):11660. doi: 10.3390/ijms252111660 (PMC11545953; doi:10.3390/ijms252111660)
Supplement: Supplementary file 1 [file ijms-25-11660-s001.zip › Figure S1.pdf]

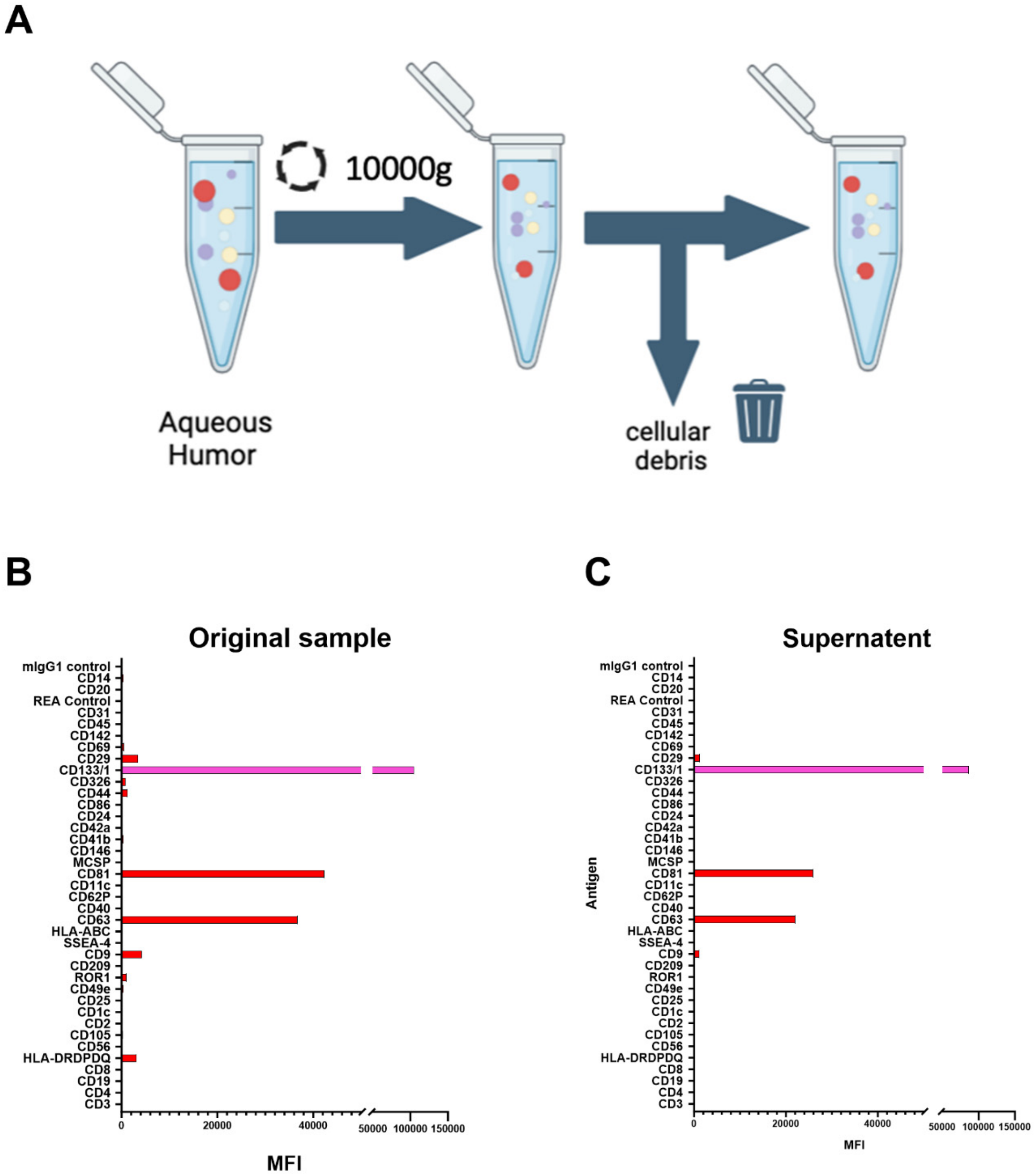

Figure S1. Surface Profile Markers of EV/EPs using MACSPlex Assay with Aqueous Humor Sample Case 79 before and after centrifugation. (A) Workflow depicting 10000g centrifugation experiment conducted on an original AH sample. (B) MFI bar plot generated using 5 μL of original AH sample from Case 79. (C) MFI bar plot generated using 5 μL of AH sample from Case 79 after 10000g centrifugation.
