# Supplementary material for: Phenotypic Biomarkers of Aqueous Extracellular Vesicles from Retinoblastoma Eyes
Source: Int J Mol Sci. 2024 Oct 30;25(21):11660. doi: 10.3390/ijms252111660 (PMC11545953; doi:10.3390/ijms252111660)
Supplement: Supplementary file 1 [file ijms-25-11660-s001.zip › Figure S2.pdf]

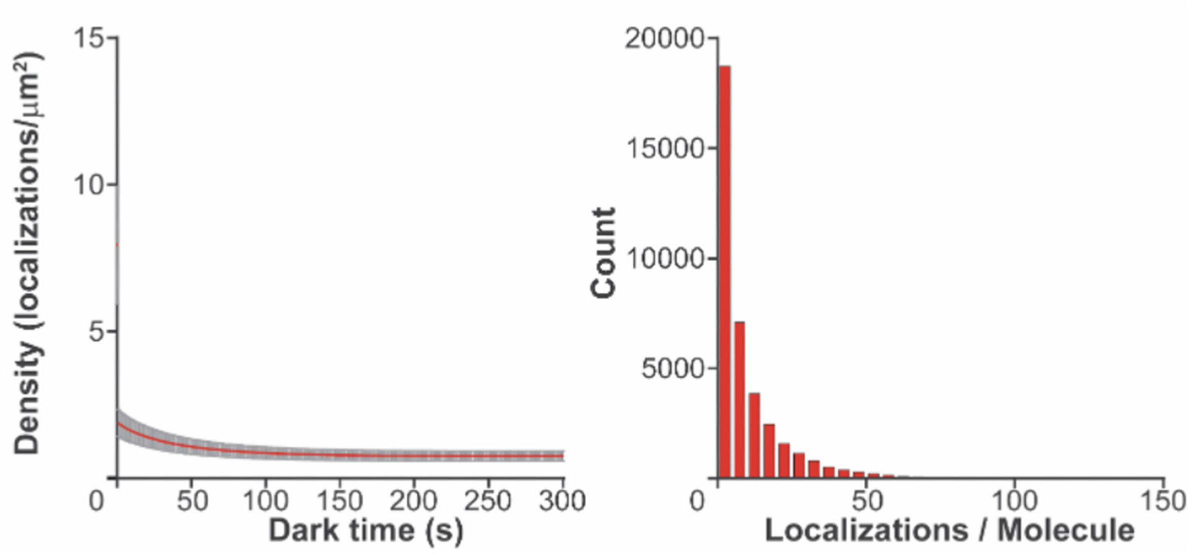


**Figure S2. Photophysical properties of AF647-labeled antibodies.** Based on 30 ROIs (from 3 independent experiments), the average number of localizations per fluorescent probe (alpha) of 11 and a maximum dark time of 250 s was obtained for the mixture of anti-CD9, anti-CD63, anti-CD81, and anti-CD133 Abs labeled with AF647.
