# Supplementary material for: Phenotypic Biomarkers of Aqueous Extracellular Vesicles from Retinoblastoma Eyes
Source: Int J Mol Sci. 2024 Oct 30;25(21):11660. doi: 10.3390/ijms252111660 (PMC11545953; doi:10.3390/ijms252111660)
Supplement: Supplementary file 1 [file ijms-25-11660-s001.zip › Figure S3.pdf]

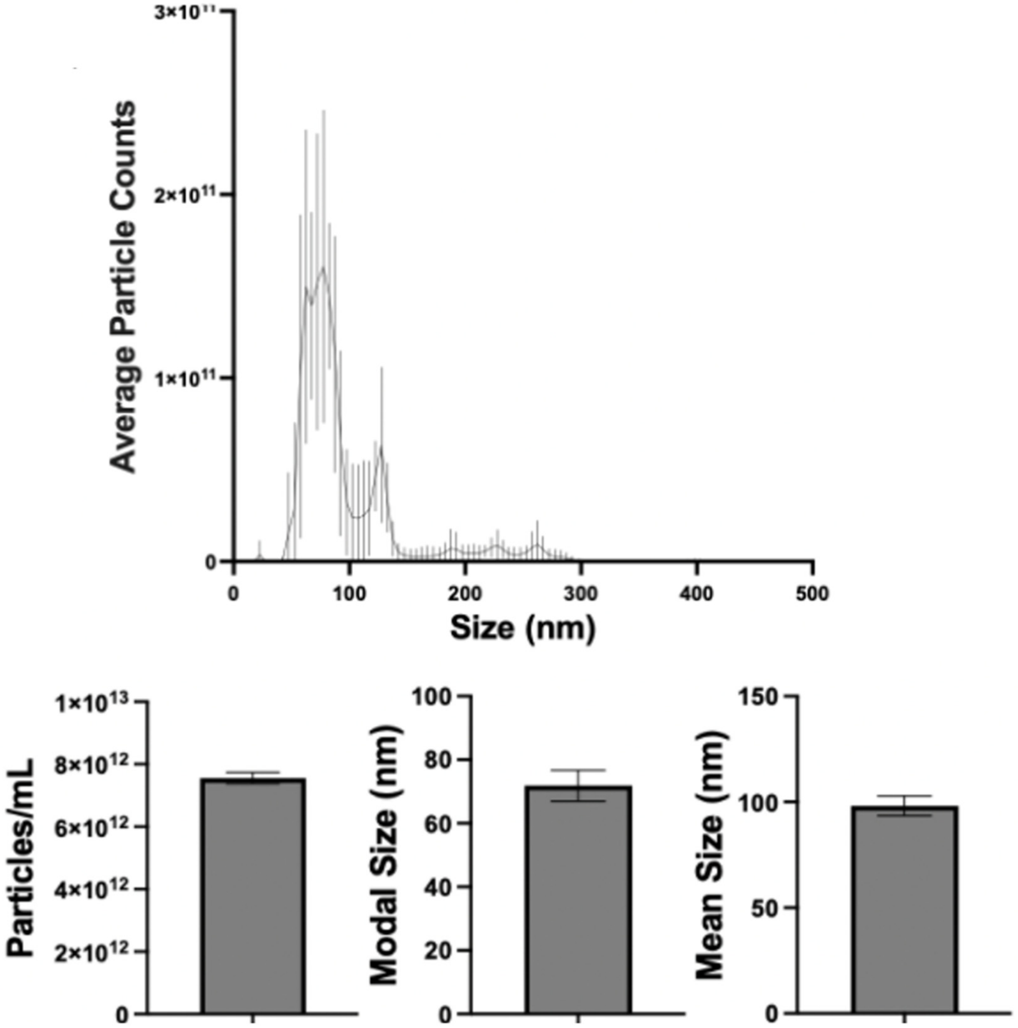

Figure S3. NTA Analysis using AH Sample from Case 79. Average particle counts per size (nm) for AH sample from Case 79. Particles/mL, modal size (nm), and mean size (nm) for particles in AH sample from Case 79.
