# Supplementary material for: Phenotypic Biomarkers of Aqueous Extracellular Vesicles from Retinoblastoma Eyes
Source: Int J Mol Sci. 2024 Oct 30;25(21):11660. doi: 10.3390/ijms252111660 (PMC11545953; doi:10.3390/ijms252111660)
Supplement: Supplementary file 1 [file ijms-25-11660-s001.zip › Figure S5.pdf]

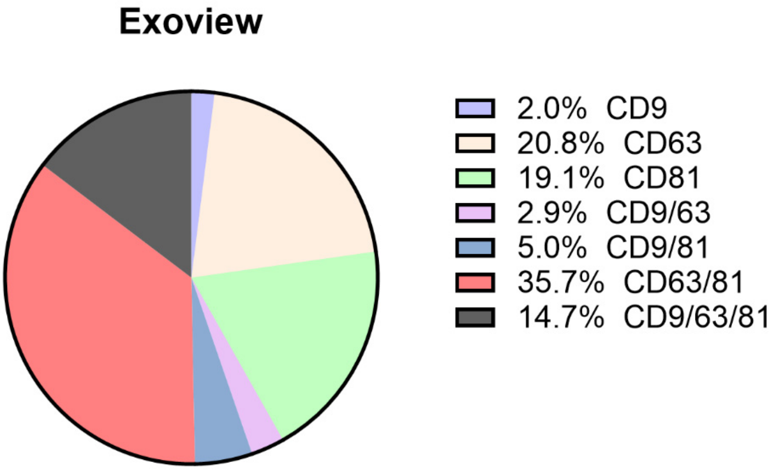

Figure S5. Subpopulation Breakdown After SP-IRIS Analysis in Aqueous Humor Sample Case 79. Percentage breakdown of subpopulations based on CD9, CD63, and CD81 positivity after SP-IRIS analysis.
