# Supplementary material for: Phenotypic Biomarkers of Aqueous Extracellular Vesicles from Retinoblastoma Eyes
Source: Int J Mol Sci. 2024 Oct 30;25(21):11660. doi: 10.3390/ijms252111660 (PMC11545953; doi:10.3390/ijms252111660)
Supplement: Supplementary file 1 [file ijms-25-11660-s001.zip › Figure S6.pdf]

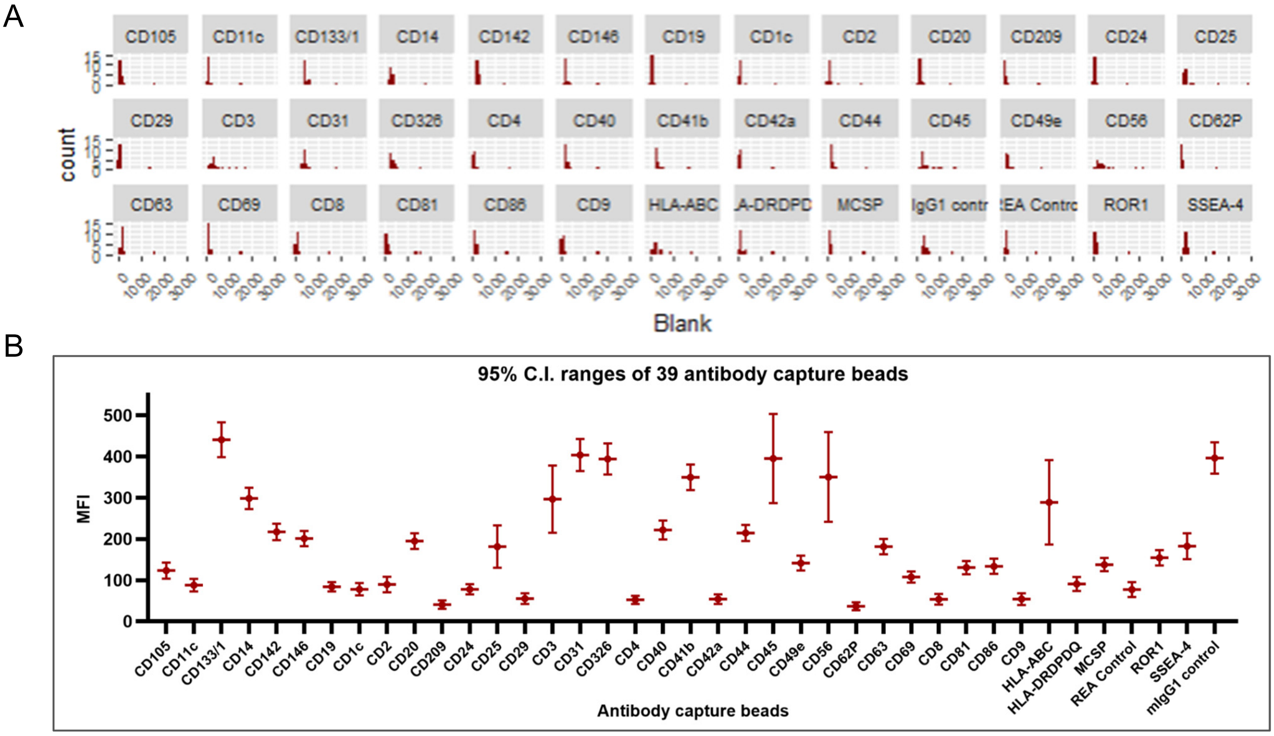

Figure S6. (A) Readout vs. count histograms created using 18 MACSPlex blank control sets, with high outliers visually evident. (B) Mean and 95% confidence interval bounds for 39 antigens using multiple MACSPlex experiments.
