# Supplementary material for: Phenotypic Biomarkers of Aqueous Extracellular Vesicles from Retinoblastoma Eyes
Source: Int J Mol Sci. 2024 Oct 30;25(21):11660. doi: 10.3390/ijms252111660 (PMC11545953; doi:10.3390/ijms252111660)
Supplement: Supplementary file 1 [file ijms-25-11660-s001.zip › Table S1.pdf]

Table S1. SEVEN data

EV count per ROI per µL						
	CD63/CD81	CD9	CD133 (TSPAN stain)	CD133 (CD133 + TSPAN stain)	IgG	No stain
Median	22500	9250	4880	27140	240	10
Mean	25873	10585	5280	25715	261	7
SEM	2079	1163	508	1374	27	2
CV	31%	49%	37%	21%	46%	123%

Diameter (nm)				
	CD63/CD81	CD9	CD133 (TSPAN stain)	CD133 (CD133 + TSPAN stain)
Median	75	77	71	71
Mean	76	80	73	74
SEM	0.3	0.4	0.3	0.1
CV	24%	25%	24%	26%

Detected molecule count/EV				
	CD63/CD81	CD9	CD133 (TSPAN stain)	CD133 (CD133 + TSPAN stain)
Median	8	9	7	9
Mean	11	11	9	11
SEM	0.1	0.2	0.1	0.1
CV	74%	85%	64%	70%

Circularity				
	CD63/CD81	CD9	CD133 (TSPAN stain)	CD133 (CD133 + TSPAN stain)
Median	0.854	0.855	0.849	0.845
Mean	0.845	0.847	0.840	0.835
SEM	0.0009	0.0012	0.0009	0.0004
CV	7%	6%	7%	7%
