# Supplementary material for: Phenotypic Biomarkers of Aqueous Extracellular Vesicles from Retinoblastoma Eyes
Source: Int J Mol Sci. 2024 Oct 30;25(21):11660. doi: 10.3390/ijms252111660 (PMC11545953; doi:10.3390/ijms252111660)
Supplement: Supplementary file 1 [file ijms-25-11660-s001.zip › Table S2.pdf]

Table S2. P-values for SEVEN data.

EV count per ROI per µL		
Dunnett's T3 multiple comparisons test	Summary	Adjusted P Value
CD63/CD81 vs. CD9	****	0.00002
CD63/CD81 vs. CD133 (TSPAN stain)	****	<0.000001
CD63/CD81 vs. IgG	****	<0.000001
CD63/CD81 vs. No stain	****	<0.000001
CD63/CD81 vs. CD133 (CD133 + TSPAN stain)	ns	>0.999999
CD9 vs. CD133 (TSPAN stain)	**	0.004
CD9 vs. IgG	****	<0.000001
CD9 vs. No stain	****	<0.000001
CD9 vs.CD133 (CD133 + TSPAN stain)	****	<0.000001
CD133 (TSPAN stain) vs. IgG	****	0.000002
CD133 (TSPAN stain) vs. No stain	****	<0.000001
CD133 (TSPAN stain) vs. CD133 (CD133 + TSPAN stain)	****	<0.000001
IgG vs. No stain	****	<0.000001
IgG vs. CD133 (CD133 + TSPAN stain)	****	<0.000001
No stain vs. CD133 (CD133 + TSPAN stain)	****	<0.000001

Diameter		
Games-Howell's multiple comparisons test	Summary	Adjusted P Value
CD63/CD81 vs. CD9	****	<0.000001
CD63/CD81 vs. CD133 (TSPAN stain)	****	<0.000001
CD63/CD81 vs. CD133 (CD133 + TSPAN stain)	****	<0.000001
CD9 vs. CD133 (TSPAN stain)	****	<0.000001
CD9 vs. CD133 (CD133 + TSPAN stain)	****	<0.000001
CD133 (TSPAN stain) vs. CD133 (CD133 + TSPAN stain)	ns	0.4

Detected molecule count/ EV		
Games-Howell's multiple comparisons test	Summary	Adjusted P Value
CD63/CD81 vs. CD9	**	0.003
CD63/CD81 vs. CD133 (TSPAN stain)	****	<0.000001
CD63/CD81 vs. CD133 (CD133 + TSPAN stain)	**	0.003
CD9 vs. CD133 (TSPAN stain)	****	<0.000001
CD9 vs. CD133 (CD133 + TSPAN stain)	ns	0.5
CD133 (TSPAN stain) vs. CD133 (CD133 + TSPAN stain)	****	<0.000001

Circularity		
Games-Howell's multiple comparisons test	Summary	Adjusted P Value
CD63/CD81 vs. CD9	ns	0.6
CD63/CD81 vs. CD133 (TSPAN stain)	****	0.00003

CD63/CD81 vs. CD133 (CD133 + TSPAN stain)	****	<0.000001
CD9 vs. CD133 (TSPAN stain)	****	0.000002
CD9 vs. CD133 (CD133 + TSPAN stain)	****	<0.000001
CD133 (TSPAN stain) vs. CD133 (CD133 + TSPAN stain)	****	0.00002

** $p<0.01$, **** $p<0.0001$, ns: not significant
