# Supplementary material for: Phenotypic Biomarkers of Aqueous Extracellular Vesicles from Retinoblastoma Eyes
Source: Int J Mol Sci. 2024 Oct 30;25(21):11660. doi: 10.3390/ijms252111660 (PMC11545953; doi:10.3390/ijms252111660)
Supplement: Supplementary file 1 [file Figure S4.pdf › Figure S4.pdf]

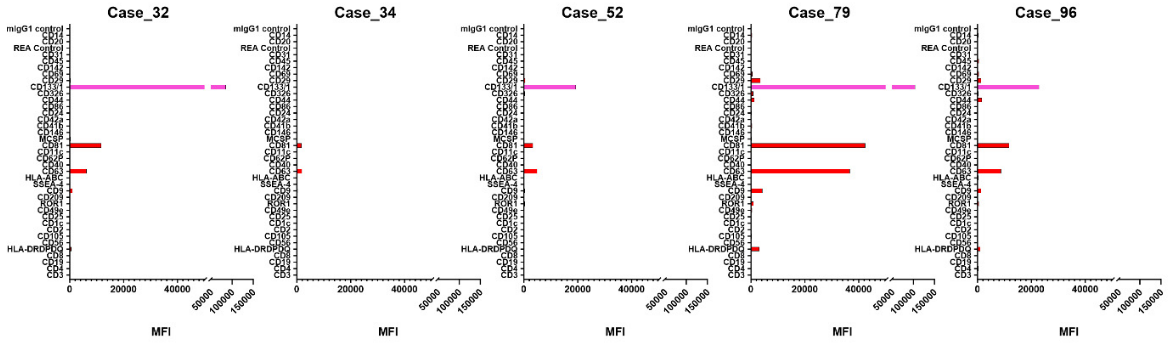

Figure S4. Scaled X-axis for MACSPlex analysis of all RB AH Samples. MFI bar plots from 5 RB AH Samples, with the X-axis scaled to 150,000 MFI.
